# Oxidative stress and metabolic diseases: Relevance and therapeutic strategies

**DOI:** 10.3389/fnut.2022.994309

**Published:** 2022-10-17

**Authors:** Muhammad Faisal Manzoor, Zaira Arif, Asifa Kabir, Iqra Mehmood, Danial Munir, Aqsa Razzaq, Anwar Ali, Gulden Goksen, Viorica Coşier, Nazir Ahmad, Murtaza Ali, Alexandru Rusu

**Affiliations:** ^1^Guangdong Provincial Key Laboratory of Intelligent Food Manufacturing, Foshan University, Foshan, China; ^2^School of Food Science and Engineering, South China University of Technology, Guangzhou, China; ^3^Department of Nutritional Sciences, Faculty of Medical Sciences, Government College University, Faisalabad, Pakistan; ^4^Department of Epidemiology and Health Statistics, Xiangya School of Public Health, Central South University, Changsha, China; ^5^Hunan Provincial Key Laboratory of Clinical Epidemiology, Xiangya School of Public Health, Central South University, Changsha, China; ^6^Department of Food Technology, Vocational School of Technical Sciences at Mersin Tarsus Organized Industrial Zone, Tarsus University, Mersin, Turkey; ^7^Genetics and Genetic Engineering Department, Animal Science and Biotechnology Faculty, University of Agricultural Sciences and Veterinary Medicine Cluj-Napoca, Cluj-Napoca, Romania; ^8^Life Science Institute, University of Agricultural Sciences and Veterinary Medicine Cluj-Napoca, Cluj-Napoca, Romania

**Keywords:** oxidative stress, metabolic diseases, a burden on health, therapeutic approaches, LDL

## Abstract

Metabolic syndrome (MS) is a prominent cause of death worldwide, posing a threat to the global economy and public health. A mechanism that causes the oxidation of low-density lipoproteins (LDL) is associated with metabolic abnormalities. Various processes are involved in oxidative stress (OS) of lipoprotein. Although the concept of the syndrome has been fiercely debated, this confluence of risk factors is associated with a higher chance of acquiring type 2 diabetes mellitus (T2DM) and atherosclerosis. Insulin resistance has been found to play a significant role in the progression of these metabolism-associated conditions. It causes lipid profile abnormalities, including greater sensitivity to lipid peroxidation, contributing to the increased prevalence of T2DM and atherosclerosis. This review aims to cover the most recent scientific developments in dietary OS, the consequence of metabolic disorders, and their most significant clinical manifestations (T2DM and atherosclerosis). It will also emphasize the effects of dietary approaches in alleviating OS in MS.

## Introduction

Metabolism syndrome (MS) is an international public health concern. Obesity, diabetes, dyslipidemia, elevated blood pressure, and hyperglycemia (1, 2). MS is highly complicated and has unclear pathophysiology (3). Numerous research back up the idea that oxidant/antioxidant imbalance may be crucial for its symptoms. Blood samples from MS patients had higher levels of indicators for OS and lower levels of antioxidant defenses, which may indicate an overproduction of oxidizing species *in-vivo* (4). Minimally modified low-density lipoprotein (MM-LDL) and “(completely or extensively) oxidized” LDL are the two primary classifications used to characterize oxidized LDL (ox-LDL) (5). The main distinction between the two categories is that while MM-LDL differs chemically from unmodified LDL, the LDL receptor still recognizes it, unlike most known scavenger receptors. However, none of the ox-LDL preparations are identified by the LDL receptor, only a variety of scavenger receptors (5). The content and the biological consequences of the many preparations that make up each of the two categories of ox-LDL vary greatly (5).

A drop in blood vitamin C and -tocopherol concentrations, a decline in superoxide dismutase (SOD) activity, and an increase in protein and lipid oxidation have all been linked to poor antioxidant defense in MS patients (4). On stopping OS in MS, several research is being conducted. According to recent studies, diets high in whole grain cereals, fruits, and vegetables and low in animal fat can improve one's overall health (6).

During the previous two decades, T2DM and atherosclerosis have become the world's leading causes of death in the last 20 years (7, 8). The prevalence of these diseases varies from region to region (9). Diabetes is linked to many other conditions and consequences leading to tissue and organ damage. The prevalence of heart diseases, including peripheral vascular disorders, high blood pressure, ischemic heart diseases, and atherosclerosis, is especially high (80%) in North American diabetic patients (9). It is also one of the leading causes of neuropathy, retinopathy, and nephropathy (10, 11).

According to the international diabetes federation (IDF), about 415 million people with diabetes live worldwide. The prevalence rate is 8.8 and 75% of people live in developing countries. By 2040, approximately 642 million people will be affected by T2DM (12). According to this survey, prediabetes and T2DM prevalence rates are 10.91% and 26.3%, respectively. Overall, 27.4 million people older than 20 years have diabetes.

On the other hand, atherosclerotic cardiovascular diseases (ASCVD) are a leading cause of global morbidity and mortality (13, 14). According to the World Health Organization (WHO), 17.9 million individuals died from cardiovascular diseases (CVDs) in 2019, 32% of all global deaths. Of this 32%, 85% were due to heart attack and stroke. The ratio of deaths due to CVDs was three quarters more in low and middle-income countries. Under the age of seventy, 17 million premature deaths occurred in 2019 due to non-communicable diseases. Of the 17 million deceases, 38% were caused by CVDs.

This paper aims to give a broad overview of OS's contribution to the pathophysiology of MS and other associated risk factors. In particular, it is focused on (i) the relationship between ox-LDL and metabolic disorders, (ii) dietary management for a reduction in oxidation and glycation of LDL, and (iii) dietary approaches to inhibit LDL oxidation and glycation. In addition, the global health burden of MS has also been discussed.

## Burden on health system

According to research in 2015, an estimated direct or indirect cost for CVDs was $555. An estimation is that annual costs will be increased to above $1 trillion by 2035 (15). In 2015, the Center for Medicare and Medicaid Services spent nearly $32,000 per capita on stroke and heart failure, almost $29,000 (16).

Diseases that occur after metabolic disturbance are linked to a process that causes LDL oxidation. Typically, 60–70% of LDL moves back to the liver after circulation, and peripheral tissues take the remaining 30–40% take the remaining 30–40%. North America, Europe, and Asia have hosted the majority of the studies on MS (17). Because of this, little is known about the prevalence and risk factors of MS in the population of sub-Saharan Africa. According to the limited studies that have been done in sub-Saharan Africa, the incidence of MS is quickly catching up to that in affluent countries (18). It could result from harmful Western food and lifestyle changes, cigarette use, and anti-HIV medication usage in those regions (19). The prevalence of non-communicable diseases (NCDs) has recently grown in sub-Saharan nations like Ethiopia due to fast economic expansion, an aging population, and sedentary lifestyles (20).

## Relationship between ox-LDL and metabolic disorders

### Diabetes

T2DM is the most common type of diabetes mellitus. It is typically characterized by chronic hyperglycemia, hyperinsulinemia, dyslipidemia, and lipotoxicity, resulting in progressive deterioration of insulin secretion and insulin action (21). Hyperglycemia results from the overproduction of free radicals, which are linked to the development of diabetes (22). It has been found that insulin resistance plays a key role in the occurrence of T2DM. Risk factors often include hyperinsulinemia, decreased high-density lipoprotein (HDL) cholesterol, elevated triglyceride, and hypertension with insulin resistance (23). Adipocyte insulin resistance and inflammation have been identified as essential factors in the occurrence of T2DM (24). It is undeniable that insulin resistance is characterized by decreased peripheral glucose uptake (primarily in the muscles) and increased endogenous glucose production. In addition, it decreased peripheral glucose utilization and impaired beta-cell function (25).

**Figure 1:**
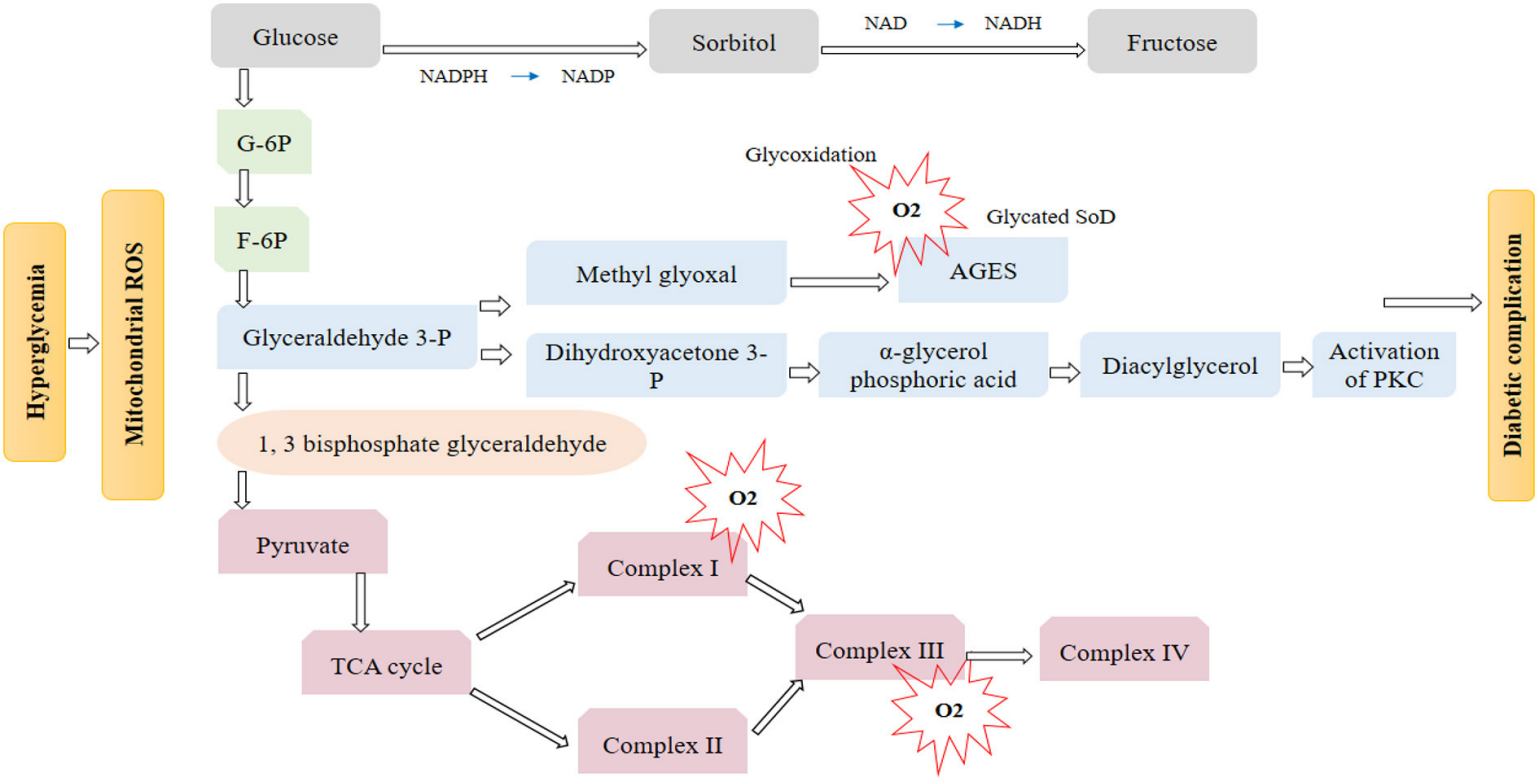
During high hyperglycemia, reactive oxygen species (ROS) is excessively increased in mitochondria. Three main pathway is responsible for hyperglycemia damage an activation of the polyols pathway, PKC pathway, and accumulation of AGES will prevent diabetic complications altogether.

Typically, in a way mentioned in Figure 1, glucose uptake, glucose moves inside the cells. But in insulin resistance, insulin receptors become resistant to insulin which ceases this mechanism, and insulin and glucose levels elevate in the bloodstream. Insulin resistance can disrupt glucose metabolism (26), resulting in chronic hyperglycemia, which causes OS and inflammatory responses that cause cellular damage. LDL is exposed to high circulating glucose levels due to the high glucose concentration in the blood. This exposure changes the LDL to glycated LDL.

**Figure 2:**
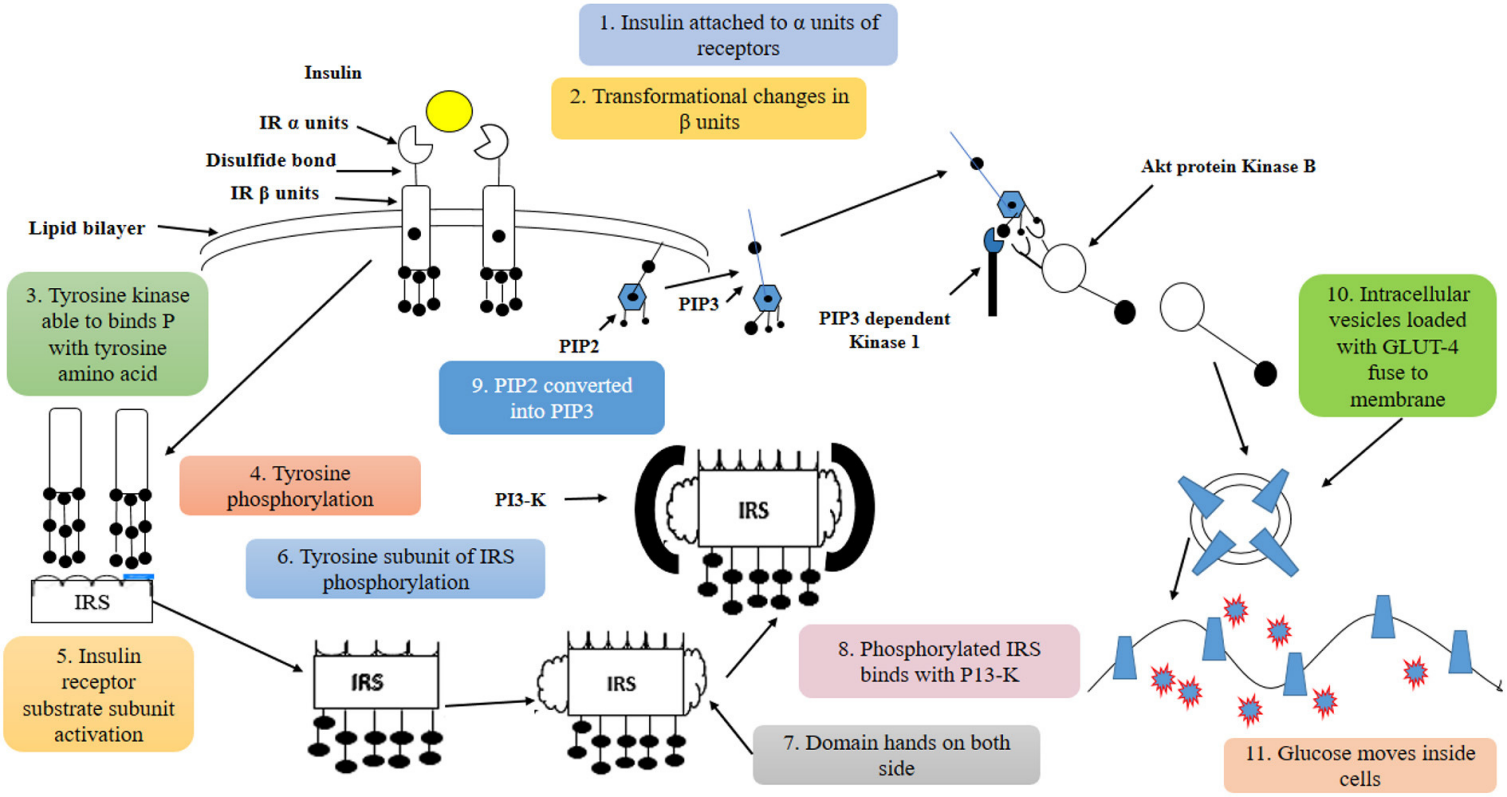
Insulin binds to insulin receptors α units which cause phosphorylation of insulin β units. After activating the insulin receptor substrate subunit, P13-K is attached to domain hands on both sides. This procedure activates Akt protein kinase B, which plays an important role in transferring glucose inside the cells.

Diabetes is also caused by excessive reactive oxygen species (ROS) produced in obese people, which causes the proliferative arrest of pancreatic beta β-cells (27). Most -cells do not have the potential to re-enter the cell cycle or have a short cell cycle length. ROS plays a significant part in the dysregulation of pancreatic cell proliferation by changing the cell cycle regulators, contributing to the onset and progression of diabetes (27). Numerous studies have also shown a direct link between elevated OS in MS and nicotinamide adenine dinucleotide phosphate oxidase (NOX) activity (28, 29) (Figure 2).

Changing dietary intake from organic healthy foods to highly processed foods may lead to increased exposure to advanced glycated end products aged garlic extracts (AGEs) by a non-enzymatic chemical reaction called glycation (30). In industries, AGEs are used to improve flavor and color and increase the shelf life of food (30). On the other hand, increased exposure to these AGEs may lead to severe health disorders. There are two types of AGE: Serum endogenous advance glycated end products (sAGE) that form within the body during digestion, absorption, and metabolism (30). Foods are the exogenous AGEs also called dietary AGEs (dAGE's). Both endogenous and exogenous AGE's significantly contributed to the total body AGE pool (31).

**Figure 3:**
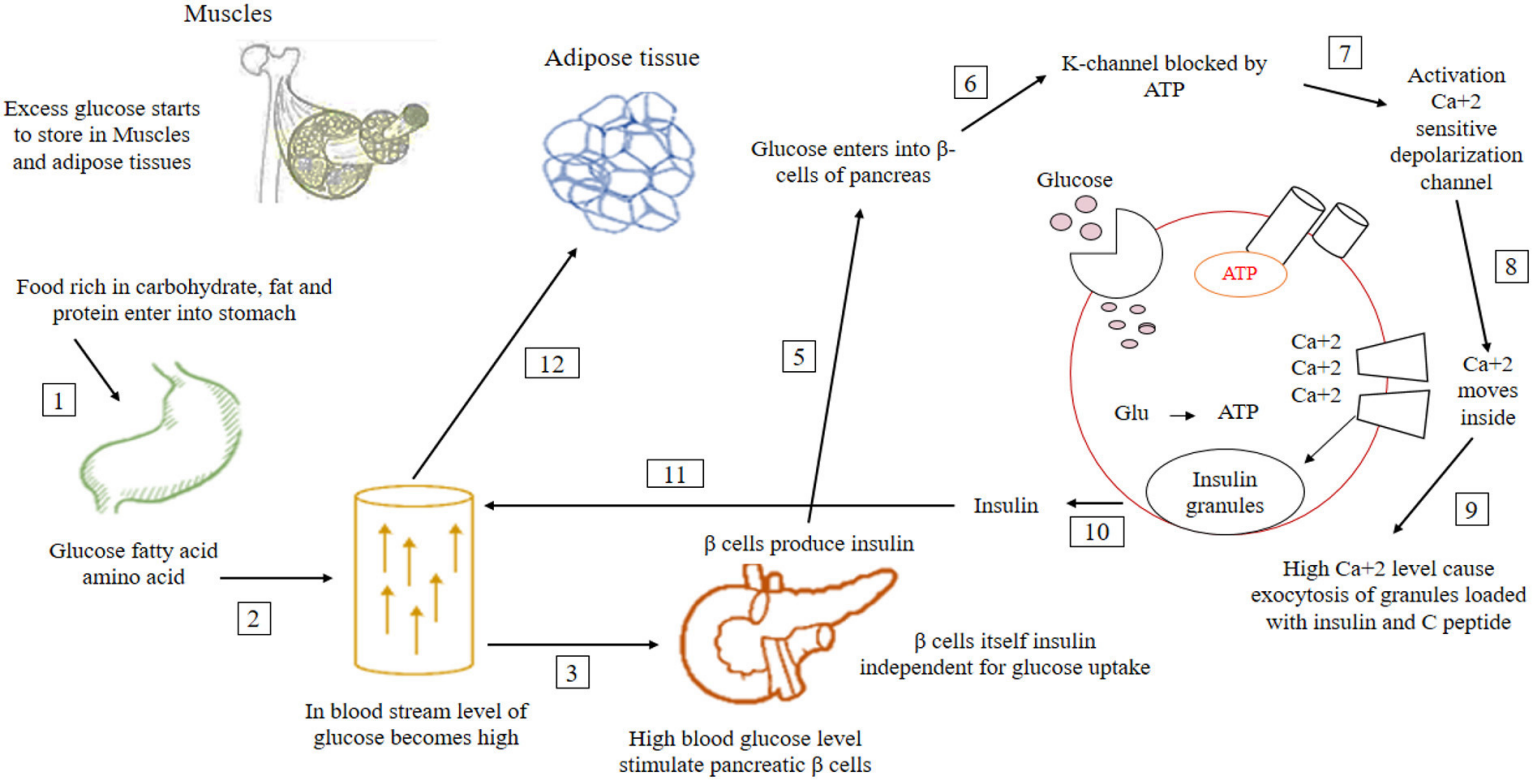
In normal conditions, after taking food, the glucose level of the blood increases. High glucose levels stimulate pancreatic β cells responsible for insulin production. After the activation of β cells, insulin level increases in the blood, which lowers glucose level to the normal range. When this insulin production is not enough for glucose utilization, glucose remains constant in the blood (35–37).

Different Researches explain that older individuals have been exposed to both endogenous and exogenous AGE (32). It leads to the development and progression of severe health disorders (32). These age-related problems are mediated and associated with OS and inflammation (33). Increased daily intake of processed foods, deep-fried foods, and high fructose items may cause inflammation and disturbance of the immune system. The primary physiological effect of insulin on nutrient utilization and intermediary metabolism occurs in the postprandial state when variable increases in plasma glucose cause insulin secretion (34). It results in glucose clearance from plasma by stimulating its uptake, using skeletal muscle and adipose tissue, and attenuating hepatic glucose production by inhibiting hepatic gluconeogenesis and glycogenolysis (Figure 3).

Insulin resistance may affect lipid metabolism as much as glucose (38). Ox-LDL was significantly linked to insulin resistance in children, young adults, and the elderly. Diabetes patients had substantially higher ox-LDL levels than non-diabetics (38). Insulin's main effects also preserve skeletal muscle mass by inhibiting protein breakdown, translating specific protein groups, and inducing lipid accumulation in adipose tissue (39). However, insulin resistance can be any impairment of insulin action on target tissues (40).

### OS in metabolic disorder leading to obesity

OS is a double-edged sword since it can cause and result in obesity. Numerous epidemiological, animal, and clinical investigations have shown that obesity and redox change are related (41). Increased OS can result from several variables, such as high-fat, high-carbohydrate diets, and persistent undernutrition, by activating intracellular pathways such as NOX, oxidative phosphorylation in mitochondria, glycoxidation, protein kinase C (PKC), and the polyol pathway (41). OS and obesity are related through mutual support pathways (42). In addition to causing a persistent chronic inflammatory state by excessive ROS formation due to a high-fat, high-carbohydrate diet and inhibited antioxidant system, obesity can also produce systemic OS through NOX activation (27). Even though it is difficult to pinpoint which comes first, inflammation and OS coexist in obesity (27). The overproduction of ROS may be further exacerbated by the redox-sensitive transcription factors, including NF-kB and activator protein (AP)-1, which are activated by ROS and produce many pro-inflammatory cytokines (27). It causes a cycle that breeds a variety of illnesses, including insulin resistance, T2DM, atherosclerosis, and cancer, all referred to as MS (27, 43).

### Atherosclerosis

Several genetic and environmental factors lead to CVDs. Oxidation of LDL is the main factor that leads to subclinical CVDs by initiating atherogenic events. These events cause the formation of mature atherosclerotic plaque. Atherosclerosis is a disease in which blood delivery and flow reduce all across the body due to the hardening and thickening of blood vessels (44).

The oxidation of LDL also aids the formation of atherosclerotic plaques. Atherosclerosis is a condition of developing complicated atherosclerotic plaques and causes the hardening and narrowing of the arterial wall (45). The Greek term atherosclerosis consists of two parts, the first one is Atherosis and the second one is sclerosis. Atherosis means fat accumulation goes along with several macrophages, and sclerosis is defined as a fibrosis layer consisting of smooth muscle cells, connective tissues, and leukocytes (46).

LDL does not directly promote atherosclerosis, but its oxidative modification in intima can increase foam cell formation at the lesion site and atherosclerotic plaque formation (47). For a clear understanding of how ox-LDL leads to atherosclerosis, there is a need to explain the whole mechanism of atherosclerosis. The atherosclerosis process includes three main steps: 1. Formation of fatty streaks, 2. Formation of atheroma, and 3. Formation of atherosclerotic plaque.

### Fatty strips formation

For a clear understanding of how ox-LDL leads to atherosclerosis, there is a need to explain the whole mechanism of atherosclerosis (48). The atherosclerosis process includes: (a): In the first step, due to elevated level of plasma LDL cholesterol, LPL-C entered endothelial cells by endocytosis. Because of the high level of plasma LDL, extracellular proteoglycans increase (49). LDL has a great affinity with this extracellular component, so it gets trapped at the lesion side in the intima wall of arteries. So, the Concentration and period of stay in intima increased (50). These factors result in spontaneous oxidative modification of trapped LDL. (b) In a second step, ox-LDL functions as an antigen for T-cells and activates T-cells, so accordingly secrete cytokines that initiate endothelial, smooth muscle cells and macrophages SMS in further steps. (c): In the third step, activated or altered endothelial cells generate adhesion molecules on leukocytes (51). Adhesion molecules have specific receptors expressing smooth muscle cells and vascular endothelial cells on specific leukocytes. In the expression of adhesion receptors, transcription factor NF-αβ is activated by pro-inflammatory binding cytokines to their receptors on the endothelial surface and performed transcription (52). These adhesion molecules play a vital role in chemokines/cytokine production and release, which is critical in the activation and release of leukocytes. Furthermore, migration of endothelial and smooth muscle cells (SMC) accurse due to specific chemokines. From various studies, it has been demonstrated that adhesion molecules are unregulated by ox-LDL. (d): In the fourth step, phagocytes differentiate into macrophages after insertion into the intima. Macrophages carry out uptake and acquisition of ox-LDL with the action of their scavenger receptors. They will convert to yellowish foam cells—cytokines and ox-LDL increase the expression of these receptors when monocytes differentiate into macrophages (51). Ox-LDL ligand surface is phospholipids that start their absorption to receptors which will be oxidized at no two locations and result in the formation of aldehydes that have the power to attack Apo lysine residues. These yellow foam cells accumulated on the walls of arteries, and lipid strips formed. Monocytes can also produce cytotoxic substances, leading to more LDL oxidation and damage (51).

### Formation of atheroma

Endothelial cells secret small peptides such as cytokines and growth factors like interleukin 1 (IL-1) and TNF causing smooth muscle cell migration and synthesized extracellular matrix. It forms the fibrous cap of collagen-rich fiber tissues, SMC, macrophages, and T-lymphocytes (46).

### Formation of atherosclerosis plaque

**Figure 4:**
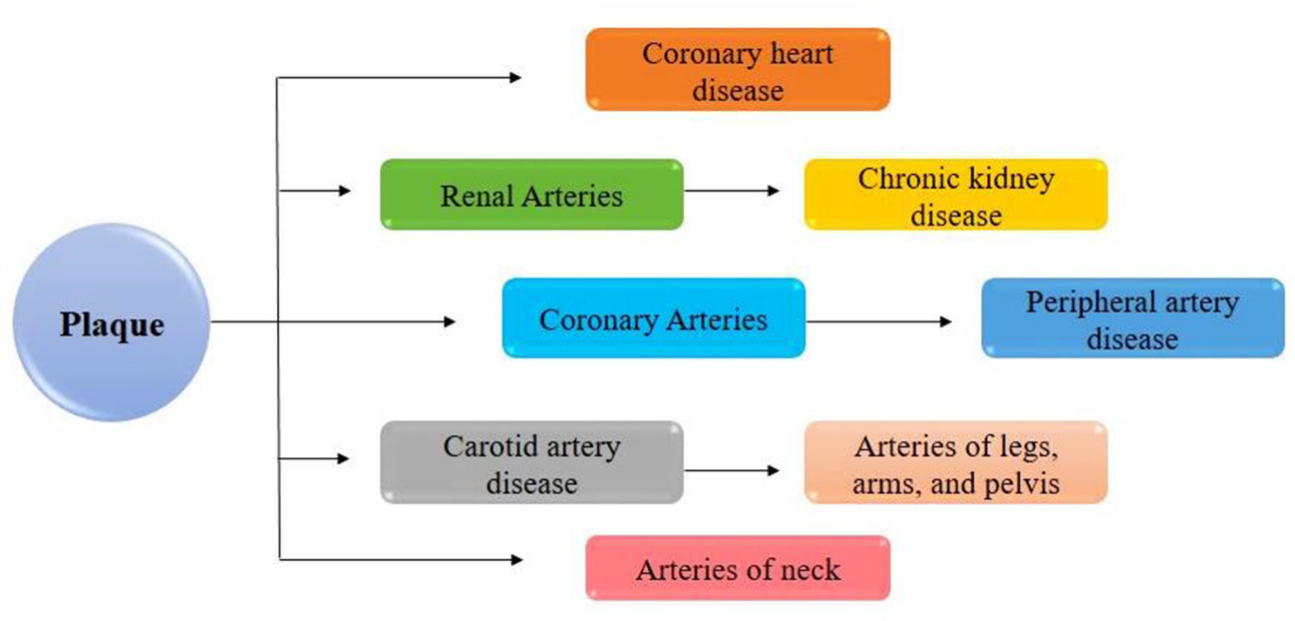
LDL is not directly promoting atherosclerosis, but its oxidative modification in intima can lead to increases in foam cell formation at the lesion site and atherosclerotic plaque formation (53).

All the above factors formed mature atherosclerotic plaque that further obstructs arteries' blood flow. So, ox-LDL can be diligently involved in the atherosclerotic process by different mechanisms, including the activation of t-cells. Endothelial cell (EC) activation and dysfunction, activation of macrophage (Figure 4). By up-regulated adhesion molecules, foam cell formation by increasing the expression of scavenger receptors of macrophages and by proliferation and migration of vascular smooth muscle cells (VSMC) (46, 54, 55).

## Dietary management for a reduction in oxidation and glycation of LDL

**Figure 5:**
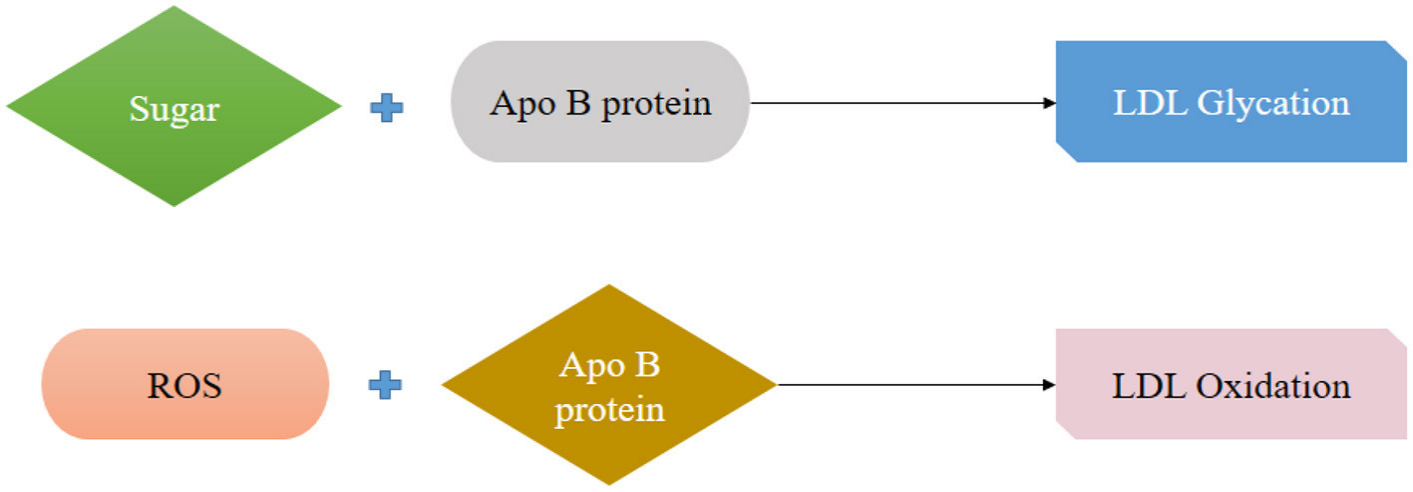
Attachment of sugar with protein strand (Apo B) is called LDL glycation, modification of Apo B by Free radicals known as LDL oxidation.

Taking foods containing complex carbohydrates like vegetables, fruits, cereals, and dairy products has a low concentration of AGEs (56). Avoid snacks, biscuits, and other processed foods as they contain high levels of AGEs. The cooking method and heating duration played an essential role in the increased production of AGEs (56). Deep frying increased the concentration of glycated products. Take adequate vitamin C, B, and phytonutrients (56) (Figure 5).

### Dietary approaches to inhibit LDL oxidation and glycation

#### Mono-unsaturated fatty acid

**Figure 6:**
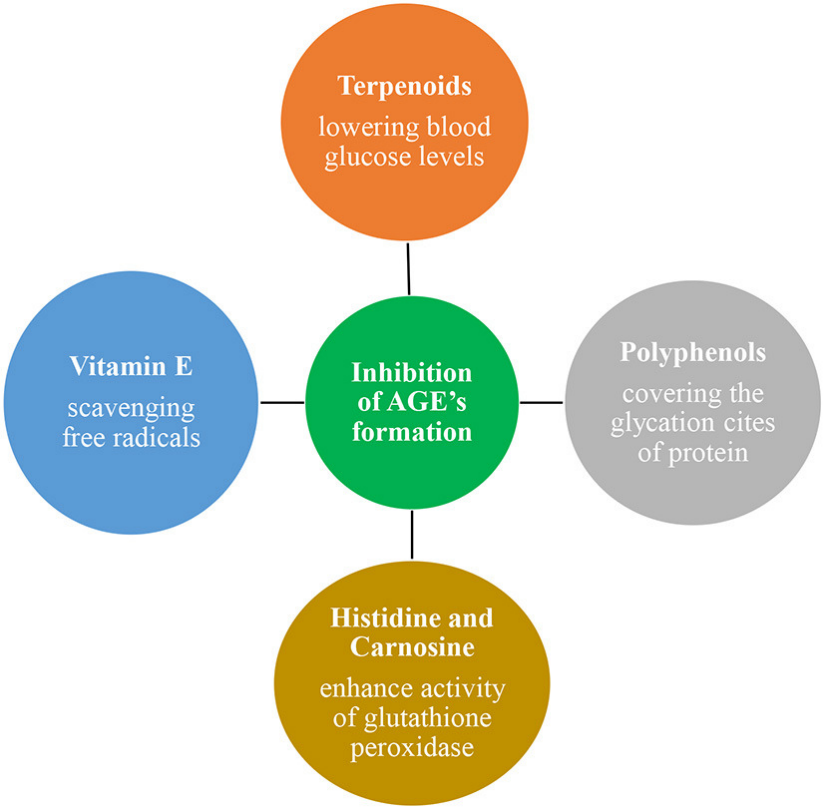
Vitamin E, Polyphenols, Terpenoids, Histidine, and carnosine have antioxidant and anti-inflammatory properties and protective roles against LDL oxidation and glycation. They protect by scavenging free radicals by covering the glycation sites of protein. And lowers the blood glucose levels.

A study was established to compare the effect of high monounsaturated fatty acid and a high carbohydrate diet on LDL oxidation (57). To reach the result, twenty men and women were taken with diabetes mellitus and a mean age of 61. They were given an isocaloric diet with carbohydrates (28% energy) and monounsaturated fatty acid (MUFA) (40% energy) for 6 weeks. After 6 weeks, LDL susceptibility to oxidation, body weight, glycemic control, and lipid profile were measured. It was concluded that both high carbohydrate and high MUFA natural food-based diets have a similar effect on LDL oxidative resistance and overall metabolic control in patients with diabetes mellitus (58). Body weight, total cholesterol and triglycerides were also the same after the two diets. Still, the only difference was that the high monounsaturated fatty acid diet lowered the very-low-density lipoprotein (VLDL) by 35% compared to a high carbohydrate diet. MUFA was also a good alternative to a high carbohydrate diet for T2DM (Figure 6).

#### High fat diet

A study showed the effect of a high-fat diet in lowering plasma triglycerides and VLDL concentration in patients with diabetes mellitus (59). This effect was due to increased lipolysis activity or increased clearance of triglyceride-rich lipoprotein (60). It was seen that HDL concentration also increased after the consumption of a high monounsaturated diet as compared to a high carbohydrate diet (61). The net increase was 0.05 mmol and reduced the susceptibility of LDL oxidation (61). According to the study, the subjects fed a diet rich in oleic acid were less susceptible to oxidation (62). Diet rich in monounsaturated acid has 27% more α-tocopherol than compared to a diet rich in carbohydrates (62). It has a protective role against LDL oxidation due to its antioxidant properties.

#### Vitamin E

Diabetes lowers antioxidant vitamin levels, making lipids more vulnerable to oxidative assault. Lipid-soluble antioxidants carry LDL like vitamin E and carotenoids (including b-carotene and lycopene) that shield it from oxidation (63). A significant water-soluble chain-breaking antioxidant, vitamin C (ascorbate), works by rebuilding a-tocopherol from its oxidized radical state (64). Diabetes patients have been shown to have lower vitamin E and ascorbate (65).

It may be due to the antioxidant property of Vitamin E, which reduces the number of oxidants and free radicals and have a protective role in lipid oxidation (66). With the admission of vitamin E, paraoxonase 1 protein has expanded in the blood, showing a decline in OS and protecting the lipids from oxidation (67).

#### Polyphenols

Polyphenols are the primary antioxidants in the human diet (68–70). They have antioxidant and anti-inflammatory properties and have a protective role against chronic health problems that involve inflammation (71–73). Culinary herbs and spices have a higher concentration of phenolic compounds and low caloric content, which is advantageous in diabetes mellitus (74, 75). One of the results of raised blood glucose is an expansion in the nonenzymatic glycation of proteins. Evidence showed that the extract of these herbs and spices might block the formation of advanced glycated end products (AGEs) (76). *In vitro* experiment showed that 50% ethanolic extracts of these herbs and spices inhibit fructose-mediated protein glycation. Extracts of cinnamon and ground Jamaican allspice are the most effective inhibitors of glycation (77, 78).

#### Histidine and carnosine

Histidine and carnosine are synthesized in the liver, skeletal muscle, and brain. These compounds are known for their antioxidant properties, such as scavenging free radicals binding the metal ions and inhibiting glycation (79). LDL oxidation and glycation result from high blood glucose (80). That cause vascular damage and further complication. Research suggests that histidine and carnosine might protect against LDL oxidation and glycation (81). Because they are amino acid-base compounds with a higher affinity for water-soluble molecules, they may compete for glucose with the apo-B part of the LDL molecule, allowing them to postpone the glycation process between glucose and the LDL protein part (82).

After the estimated time, ingestion of histidine and carnosine at a ratio of 1 g/L in diabetic mice significantly reduced blood glucose and fibronectin levels (81, 83). These agents showed a dose-dependent effect in suppressing malondialdehyde formation and glycation (81). Treatments with 1 g/l histidine and carnosine significantly enhanced glutathione peroxidase activity (81). In diabetic mice, consumption of histidine or carnosine greatly reduced the activity of interleukin (IL) 6 and tumor necrosis factor (TNF) alpha (84).

#### Garlic extract

In different studies, aged garlic extract inhibited LDL oxidation and reduced oxidized-induced cell injury (85, 86). The antioxidant effects of AGE were investigated further using bovine pulmonary artery endothelial cells (PAEC) and murine macrophages (86). Lactate dehydrogenase (LDH) release and intracellular glutathione (GSH) levels were measured as indicators of membrane injury. Ox-LDL increased LDH release while depleting GSH. These changes were prevented by pretreatment with AGE (86).

#### L-carnitine

L-carnitine protects against CVD by increasing HDL cholesterol, inhibiting LDL cholesterol oxidation, and neutralizing the atherogenic effects of ox-LDL cholesterol (87). Reduced concentrations of TBARS (Thiobarbituric acid reactive substances) and conjugated dienes, which are indices of lipid peroxidation, in the blood of diabetic hyperlipidemia patients (87). These lower concentrations could be attributed to a decrease in or increase in the use of antioxidant mechanisms (87). Changes in the composition of LDL cholesterol may result in conformational changes, resulting in a different exposure of fatty acids to oxygen-free radicals and changes in the rate of lipid peroxidation (88).

#### Novel strategies

New combination approaches have been used to target glycolysis (through targeting PKM2 or pyruvate dehydrogenase kinase) and increasing oxidative phosphorylation, resulting in increased OS in cancer cells (89, 90). Different studies found that inhibiting de-novo lipogenesis in prostate cancer cells with soraphen A (an inhibitor of acetyl Co- carboxylase) causes an increase in polyunsaturated fatty acids, OS, and greater sensitivity to chemotherapeutic treatments (91). Overall, OS is integral to carcinogenesis and cancer cell metabolism and presents novel treatment possibilities (91). Phytochemicals like green tea component epigallocatechin-3 gallate and turmeric component curcumin have been shown to reduce obesity-associated polyp formation in animal models by inhibiting PI3K/Akt and MAPK signal pathways and have been suggested as a prevention strategy for obesity-associated colon cancer (92). Combination therapies target glycolysis (by targeting PKM2 or pyruvate dehydrogenase kinase) and encourage oxidative phosphorylation, leading to increased OS (93). Antioxidant and anti-inflammatory effects of curcuminoid-piperine combination in subjects with MS have also been studied in randomized control trials (94).

## Lifestyle modification

Lifestyle factors such as smoking, drinking alcohol, eating a proper or improper diet, exercising, and being untrained all contribute to OS (95). According to different studies, ROS exists in muscles and controls muscle function (96). ROS are continually produced at low levels by skeletal muscle fibers, and these levels rise during muscular contraction. They are implicated in skeletal muscle exhaustion during intense exercise and have several direct and indirect impacts on muscle function (contractility, excitability, metabolism, and calcium homeostasis) (96).

Restoring the body's redox equilibrium is one of the most acceptable ways to eliminate harmful OS (97). The objective may be to regain a healthy BMI through physical exercise and a low-fat, low-carbohydrate diet rich in antioxidants (98). A clinical investigation found that weight loss reduces signs of OS and boosts the antioxidant system, lowering the risk of CVDs linked with obesity (99). Natural fruits (100), green vegetables (101), whole grains (102), legumes (101), fish (103), olive oil (104), and probiotics (105), which are high in MUFA and polyunsaturated fatty acids (PUFA), vitamin C, vitamin E, and phytochemicals, aid in weight management and reduce the risk of metabolic diseases *via* a variety of mechanisms including cell signaling, altered gene expression, and decreased OS (101). Physical activity and exercise improve the body's antioxidant system, which aids in the management of OS by scavenging harmful free radicals, and alters cell-signaling pathways, which activate detoxification enzymes, reduce inflammation, promote normal cell cycle, inhibit proliferation, induce apoptosis, and prevent tumor invasion and angiogenesis (101).

## Conclusion

OS is linked to all modern diseases. Diabetes, CVDs, and cancer are the top causes of death worldwide. These disorders are brought on by OS, which causes inflammation. LDL is oxidized, forming AGEs and ox-LDL end products, damaging the cellular mechanism and disturbing function. This damage results in the development of diseases. Dietary oxidation is a significant cause of ox-LDL and AGEs, which can be addressed through appropriate diet management and consumption of suitable phytonutrients. Since OS has become a major factor in chronic metabolic diseases, it is crucial to: (i) further understand the mechanisms that disturb the healthy balance between oxidative and antioxidative processes; (ii) incorporate various nutritional antioxidants into current therapies, including those that can neutralize OS, such as flavonoids, arginine, vitamin C, vitamin E, carotenoids, resveratrol, and selenium; and (iii) prevent mitochondrial dysfunction from boosting defenses against OS.

## Author contributions

Conceptualization: MM, NA, MA, and ARa. Writing—original draft preparation: ZA, AK, IM, DM, GG, ARa, AA, and MM. Writing—review and editing: MM, NA, MA, and ARu. Revision of article: VC, MM, and AA. Supervision: MM and ARu. All authors have read and agreed to the published version of the manuscript.

## Funding

This study was supported by a grant from the Romanian National Authority for Scientific Research and Innovation, CNCS-UEFISCDI, Project Number PN-III-P2-2.1-PED-2019-1723 and PFE 14, within PNCDI III. The authors also want to acknowledge the support of Guangdong Provincial Key Laboratory of Intelligent Food Manufacturing, Foshan University, Foshan 528225, China (Project ID:2022B1212010015).

## Conflict of interest

The authors declare that the research was conducted in the absence of any commercial or financial relationships that could be construed as a potential conflict of interest.

## Publisher's note

All claims expressed in this article are solely those of the authors and do not necessarily represent those of their affiliated organizations, or those of the publisher, the editors and the reviewers. Any product that may be evaluated in this article, or claim that may be made by its manufacturer, is not guaranteed or endorsed by the publisher.
